# Practitioners’ perspectives on family-based intervention for illicit drug-using adolescents in Taiwan: a qualitative study

**DOI:** 10.1186/s13011-022-00460-8

**Published:** 2022-04-26

**Authors:** Lanying Huang

**Affiliations:** Graduate School of Criminology, National Taipei University, New Taipei City, Taiwan

**Keywords:** Adolescents, Family-based intervention, Illicit drug, Harm reduction

## Abstract

**Background:**

Drug-related psychiatric comorbidity or death among adolescents has grown to become a public health threat in Taiwan. In an atmosphere of abstinence, few alternatives or rehabilitative options for troubled young people have caused many juveniles to be driven away from home and placed in closed institutions. The intersectionality of illegal substance use, deviant behaviours, and criminal offences among adjudicated adolescents is a challenge for the development of adolescent users’ harm reduction strategies. In addition, the relationships among the adolescents, their families, and practitioners might be undermined by coercive and mandatory court measures. As developing a harm reduction approach includes minimizing the negative effects on not only adolescents and also their families, this study aims to explore Taiwanese practitioners’ perspectives of family-based programmes for drug-using adolescents.

**Methods:**

This study adopted a qualitative approach. Through face-to-face interviews and a focus group, accounts were collected from 28 key informants working in youth delinquency. The interviews were transcribed for analysis using qualitative analysis software, namely NVivo10.

**Results:**

Practitioners tend to refer to an “adolescent-centred model” when describing their work with drug-using young people. From the frontline practitioners’ descriptions, the families of those youth needing treatment services are often vulnerable and disadvantaged. These families are seldom onboard because of low readiness and scarce resources. Although a legal framework is in place, resources, workforce, and programmes to engage and involve parents in the intervention are lacking. Despite the obstacles, practitioners have utilized a variety of strategies to obtain limited success, such as parent meetings, resources referrals, parent counselling, court support, voluntary parenting courses, illicit substance information sharing, and home visits. Practitioners also pointed out that therapeutic interventions are more effective than coercive or mandatory interventions for adolescents and also for their families.

**Conclusions:**

Since most practitioners have an adolescent-centred work principle, their work with parents falls into professional-centred or family-allied work models. It is therefore suggested that resources be reallocated to involve parent counselling and intense treatment instead of mandatory parental education.

## Background

### Illicit drug-using among adjudicated adolescents in Taiwan

Drug-related psychiatric comorbidity or death among adolescents has grown to become a public health threat in Taiwan. From 2013 to 2017, a total number of 2,029 drug-related deaths was recorded by the Institute of Forensic Medicine in Taiwan, which is 2.8 times the number of homicide victims [1]. Among them, more than 10% (216) victims were below 24 years old. Death of illicit drug toxicity has become the top post-mortem autopsy death cause for children (below 14 years old) and young people (between 15 and 24 years old) in Taiwan.

Taiwan, like many other places in the world, has emphasized abstinence goals in terms of drug policy for both adults and adolescents. Within this abstinence context, Taiwan is adopting a dual system approach, namely social welfare, and juvenile justice, combining deterrence and protection measures to deal with adolescent drug use. The Statute for the Prevention and Control of Illicit Drugs (renamed in 1998 from the Narcotics Hazard Prevention Act 1955) represents the traditional mainstream deterrence and punishment perspective. The Protective of Children and Youths Welfare and Rights Act (2013~) has provisions to protect children and youth from the harm of substances such as reporting obligations of first responders and the responsibilities of parents as guardians. The Juvenile Delinquency Act (1962~) rules using the non-listed substance as status offences and judicial protective measures for using listed substances. Since the minimum age of criminal responsibility is 18 years old, those who are between 12- to 18-years old or who are under 12 years old but referred to the court will be dealt with in a separate juvenile tribunal.

According to police records, the recorded number of illicit drug-using adolescents has increased from 2000 and peaked in 2015 (1,939 persons) [2]. In 2019, illegal substance use is the fourth reason for youth violations of laws, following theft, fraud, and assault. Between 2015 and 2017, nearly one in every five recorded juvenile offences is illegal drug-using. Previous survey studies on pre-trial detained youths found the prevalence rate of illegal substance use varies from 64.6 to 72.2% [3–5] while the prevalence rate of other survey studies based on campus population is below 2% [6, 7]. Ketamine and Methamphetamine are the most frequently used substances among adolescents and growing popularity of coffee-milk tea mixed drink pack, which often contains mixed polydrug, is also noted in recent years [6, 8, 9]. The intersectionality between illegal substance use, deviant behaviours, and criminal offences among adjudicated adolescents is a challenge for developing adolescent users’ harm reduction strategy in Taiwan.

### Adolescents, families, drugs, and intervention

The vast number of adjudicated youths are subject to protective orders in Juvenile tribunals. Corrective education is supposed to be the last resort and applied to those adolescents who cannot benefit from other protective measures. Nevertheless each year from 2012 to 2017, more than 200 adolescents were sent to reform schools for illegal substance use. At the same time, a total of 4,820 adolescents were detained for pre-trial investigation and 10,410 were sent to Juvenile Tribunals as status offenders for unregulated substances misuse [2]. Using illegal and unregulated substances has become the main reason for adolescents to be placed in detention or corrective education institutions.

Too few alternative or rehabilitative options exist for troubled young people has caused many juveniles to be sent away from home and being placed in closed institutions in Taiwan. The number of youths in detention for illicit substance use peaked in 2013 at 1,067, which is one in four detained youths. A few qualitative studies have tried to describe the dynamics between adolescents, the juvenile tribunals, and the parents of adjudicated youth [10–12]. It has been found that family is an important consideration for a judge’s placement decisions [12]. Although youths who are detained usually have weaker family support, sometimes the parents will ask the judge to order their children into reform education facilities [13]. As the decreasing use of punitive detention is the goal of the 2019 revision to the Juvenile Delinquency Act, it brings the long-standing lack of family programmes in schools, juvenile courts, and communities to the surface. As developing a harm reduction approach include minimizing the negative effects not only for adolescents but also for their families, this study aims to explore Taiwanese practitioners’ perspectives on family-based programmes for drug-using adolescents [14].

### Literature review

#### Young people and illicit substance use

According to the World Drug Report 2018 published by the UN, 12–17 year-old adolescents go through a critical risk period of substance use [15]. Substance users who had their onset experience at earlier ages are more likely to become chronic abusers [16] or to develop more problematic behaviour [17–19]. In sum, during the highest risk stage as an adolescent, the development of criminal offending and harmful use of illicit substances are intertwined. More effort is needed to reduce the health risks and decrease the chances of substance elevation, polydrug use, and related issues [19–21].

#### Family-included treatment for adolescent drug users

In reviewing the literature, several key elements in effective treatment programmes for adolescent substance users can be identified, including multi-dimensional treatment, therapeutic intervention, and treatment that follows a risk-need-responsivity (RNR) principle [22–26]. In terms of risk identification, a chaotic home environment and ineffective parenting are among the key factors that affect both youth’s early onset and his or her trajectories from onset to harmful substance use [15, 27]. A recent cross-sectional survey in Europe shows that the risk of substance use was highest with four or more Adverse Childhood Experiences (ACEs) [28]. This suggests that programmes that solely target adolescents have many limitations since the behaviour of minors is formed in the family environment in which they reside. The importance of the family element for effective programmes has been emphasized [20, 29, 30]. Although the term family usually refers to a wider range of cohabiting members, parents are usually the key persons who make up the adolescents’ family. Therefore, the following discussion will use “family” and “parents” interchangeably.

For adolescents, harm reduction interventions usually include family care plans in addition to individual care [31]. In England and Wales, the Independent Commission on Youth Crime and Antisocial Behaviour has identified family programmes as key for youth crime prevention and restoration [32]. In Australia, the New South Wales Ministry of Health also stresses family-centred programmes since the family is the primary source of information and ongoing resource for treatment plans [33, 34]. Family-included treatment deals with not only substance use issues but also developmental or mental health problems, which might be the result of family environment and family dynamics. Working with families is also considered to be a preferable method for engaging involuntary clients such as substance-using adolescents [34].

#### Family-included treatment is evidence-based practice

Family-included treatment is evidence-based since empirical studies show that: firstly, programmes targeting family are better than those targeting individuals; secondly, family-included therapeutic treatments improve the effectiveness and satisfaction with the treatment [35]. For young substance users, family therapy is widely used and shows better treatment effects [30, 36–38]. In sum, treatment projects which include family members or simply support family members can result in better prevention and more effective treatment  [34, 39].

#### Obstacles of engaging families in treatment programmes

In the guidebook by Vincent et al. [40], parental involvement is one of the responsivity factors in the RNR principle which might affect a youth’s ability to make progress in interventions. Therefore, intervention practices ought to consider both identifying and changing poor parenting practices and improving parental involvement. After all, engaging and maintaining families in an adolescent’s intervention services can face many obstacles [41, 42].

First of all, the characteristics of targeted families make them hard to reach and even harder to work with. These family environments are characterized as high risk which makes the adolescent a so-called crossover youth, meaning young people who are involved with both the juvenile courts and the child welfare system [43]. Many scholars have summarized the parental risk factors which are associated with an adolescent’s substance use, such as experience and attitudes to illicit substances, poor parenting, domestic violence, and lack of socialization for development [44–46]. A Taiwanese social worker C. Y. Lin once described families with delinquent juveniles as “chaotic” [13]. In Lin’s study, parent-child relationships in already unstable families are characterized as emotionally distant, having negative interaction, blurred boundaries, and full of conflict. Of all the risk factors, a parent’s substance use or a parent’s failure to disapprove of substance use have been a strong predictor of adolescent substance use [47, 48].

The changing interactions between parents and adolescents during adolescent involvement with substances and thus the justice system can make intervention difficult. The parent-child relationship might go from bad to worse when children begin to use substances. In Taiwan, Lin discovered that substance-using adolescents report acting violently toward their parents during domestic conflicts [13]. A small sample study of substance-using families in Taiwan found that these families report suffering from depression and anxiety [49]. A survey of parents of delinquent adolescents also found that they expressed needing assistance in family economics, addiction rehabilitation, job seeking, family communication, and parenting skills [10].

The interactions between parent and child become more complicated when the case is submitted to the court. Lin’s study [13] describes the contacts between parents and the criminal justice system as humiliating, burdensome, feelings of powerlessness, and stress. Unresolved family risk factors continue to affect adolescents even during the probation period or after they leave reform education institutions. In a literature review article, Anthony et al. [50] pointed out that a critical reason for the re-admission of formally incarcerated youth is returning to family members with limited ability to intervene. Qualitative interviews with judicial youths in Taiwan also found that poor communication between juveniles and their parents might cause re-offending and re-admission into reform education institutions [51]. In contrast, if the relationships between parents and their formally institutionalized youths improve, the young people are more likely to stay at home, which prevents them from re-offending [12].

#### Family preservation model, family decisions, and collaborative family work

Family preservation programmes have been widely utilized in child protection, multi-system treatment in juvenile justice and mental health to strengthen families and prevent removal, child maltreatment, and delinquency [52]. One of the best-known service models is family group conferencing (FGC) in New Zealand. To change the dynamics between adolescents, families, and the judicial system, New Zealand’s Children, Young Persons, and Their Families Act 1989 includes FGC in the case process for young people between 14 and 17 years old. FGC aims to place family and community instead of courts at the core of assisting children and young people to develop socialized behaviours which meet their social and psychological needs [53]. FGC can provide more support for young people and their families while using more community treatments and involving Maori people in the decision-making. In the Maori language, WhÃnau Ora (Family health) aims to create and maintain a positive and healthy lifestyle. Sanctions are made in FGCs to improve the development of children in the family [35].

Youth justice workers in Australia and the UK have applied a six-step problem-solving model to delivering collaborative family work to children and adolescents in the criminal justice system [54]. Trained juvenile justice workers provide collaborative family work in six steps: role and ground rules, identifying problems, deciding priorities, goals, exploring problems, and strategies. In New South Wales, collaborative family work is provided as part of the routine offerings of intervention and has proved effective in reducing recidivism [55, 56]. Workers in New South Wales responded that this service improved family communication and helped the family to solve problems that concerned them the most.

### Objective

The design of the juvenile justice system in Taiwan has provided the setting for the family to engage in the intervention of adolescents with substance use problems. The Juvenile Justice Act emphasizes the role and responsibility of parents in caring for and disciplining their children. However, studies by Taiwanese scholars have also pointed out that while emphasizing court as the last resort, scarce support is available for parents whose children might need treatment. Therefore, it is wondered if the current system has adopted any family-included intervention for substance-using youth. If any, what treatment model lies beneath the current practice? Secondly, the majority of literature in other countries focuses on analysing the effectiveness of family projects. Nevertheless, it has been found that getting parents involved in the process is challenging. This current study will explore, firstly, the views of practitioners regarding families and family-cantered treatment; secondly, the process and strategy of including parents in the treatment process; and thirdly, the opportunities and difficulties frontline practitioners face while trying to involve parents.

## Methods

This study adopted a qualitative approach using interviews and focus groups to collect data in the field. The researcher was interested in understanding the actions, decisions, beliefs, and values, of front-line practitioners rather than building a cause-effect model [57]. Accounts were collected from 28 key informants working in youth delinquency through face-to-face interviews and a focus group. The study used purposeful sampling to choose representative interviewees. A list of candidate interviewees was developed at an early stage of fieldwork. A total of 35 persons or institutions were contacted through letters, emails, and follow-up telephone calls, and seven refused to participate. All interviews were conducted during December 2018 and January 2020, and a focus group (with Worker 10, Judge 1, Advocate1, and 2) was held in May 2020. The interviews collected experiences and views at the practice level, and the focus group invited stakeholders from different backgrounds at the policy-making level to review and audit the provisional research findings and suggestions. The reason for conducting a focus group was to facilitate debates and advance the density and depth of analysis. On the other hand, one-on-one interviews with practitioners allowed them to remain confidential and to talk freely. The sampling of the informants targeted a variety of governmental and non-governmental organizations. The informants cover practitioners from schools, police, social departments, juvenile courts, and juvenile correction departments. The social workers were oversampled because five of them are based in two different local governments and another five workers are based in five different non-profit organizations as contract service providers. My data collection ended in May 2020 for two reasons: first of all, all of the potential informants successfully contacted were interviewed; and secondly, no new information emerged from new interviews. All data collection was conducted by the researcher alone, who has had three years working experience as police officer and 17 years as university faculty member and who has conducted qualitative research in the last two decades. All of the informants voluntarily participated in this study with a signed consent form. This study was conducted with the approval and supervision of the Research Ethics Committee of National Taiwan University (201807ES016).

The interview outline includes three sections: firstly, first-hand observations of the current youth substance use situation; secondly, work experience with the young people who use illicit substances and their families, and especially their parents; and thirdly, the reflection of the drug policy and strategy focusing on overall treatment and family-included treatment. To reduce the possible social desirability bias, firstly, all of the questions were open questions to minimize demand effects; secondly, each participant was given a code according to their profession to ensure their confidentiality [58, 59]. So Police 1–4 are juvenile police interviewees and workers 1–10 are youth social workers from non-profit organizations and juvenile counselling committees. One social welfare department supporting staff member is coded as SW1. Caseworkers in the Health Department are coded as Health1-3. Juvenile probation officers including a Juvenile Investigation Officer and a Juvenile Protection Officer are coded as Probation1 and Probation2. School teachers in the counsellor’s office are coded as School1-4. A juvenile correction facility officer is coded as Correction1. Two youth advocates are coded as Advo1 and Advo2. Finally, a Juvenile tribunal judge is coded as Judge1. Each interview/meeting lasted between 1 h 17 min and 3 h 15 min. The researcher kept a research diary after each interview. All of the interviews were recorded and transcribed for analysis using qualitative analysis software, namely NVivo 10.

Since the current study intended to explore the state of the art of family-based intervention for drug-using adolescents, I adopted thematic analysis to categorize and label the data [57, 60]. Thematic analysis has the strength to create themes based on the deepened understanding of the experience, perceptions, and values of practitioners. After uploading all the interview transcripts to the software, the researcher read all the text and created nodes for labelling the relevant text (open coding). Secondly, a research assistant was asked to code 10 transcripts in NVivo 10, crosschecked with the initial coding framework. These coding results were discussed, reviewed, and compared to refine and crystallise the coding framework. Thirdly, a new coding structure was used to recode all 28 transcriptions. Each node was reviewed and refined back and forth until all nodes were reorganized and grouped into five categories (tree nodes): description of family relationship (12 nodes), experiences of working with family (13nodes), obstacles (2 nodes), challenges (11 nodes), and family service suggestions (10 nodes) (see Table 1).

**Table 1:** Coding structure

Themes	Nodes	
Family relationship	Hard to reach, dysfunctional, substance-using, passive, unaware, actively seeking help, mixed feelings, hard to change, harsh parenting, middle class, criminal, poor communication	
Experiences of working with family	making contact	home visit, empathetic conversation
	Supporting the family	resources referral, parent meetings, court service, substance information
	Family intervention	parent counseling, voluntary parenting education, family support group, parent and children group, mandatory parenting education, different social workers for youth and family, parent and children meetings
Obstacles	Mandatory sanctions weaken the family Ineffective mandatory parenting education	
Challenges for family programmes	Juvenile-centered principle, few resources, project sustainability, family-based project does not work, parental consent, court decision excludes family, too many resources for ineffective treatment, service dilemma, legitimacy of service, parenting education for disabled people, lacking evaluation tools	
Family service suggestions	More family counseling and less mandatory education, more family-based projects, parent obligations, family decision making, family preservation programmes in institutions, focusing on vulnerable family, family therapy, improving the quality of mandatory education, parent notification for 3rd or 4th grade listed substance use, graded family programme options	

## Results

The results are outlined in four sections: firstly, the description of family from the practitioners’ point of view; secondly, the practitioners’ attitudes and opinions on family work; thirdly, the work strategy used to contact, support, and intervene the family; and finally, the problems and feedback from their experience.

### Attitudes of the parents

 This current study found that parents can be characterized into five main types: those who are hard to reach; dysfunctional parents; those who give up out of frustration; those who do not disapprove of using drugs; and, finally, those who want to intervene but do not know how.

#### Hard to reach parents

When asked about the role of the family in their work with problematic drug-using youth, the responses are usually negative. Thirteen sources described the parents as irresponsible for several reasons, such as being unavailable, neglectful, or distant. The practitioners mentioned that some parents are usually absent from the family and could not be reached by the children or by the practitioners.

There is one family that the kid seldom sees the parent (father) because the father is with his girlfriend. (School1)

Before (the hearing) the parent was there. But after the judicial verdict was made, the parent might be hiding from debt, or behind bars, etc. (Probation2)

You don’t see the parent on a home visit. You just see the child left at home alone. The parent is never there. (Worker6)

#### Dysfunctional parents

Before the practitioners contact the adolescent, the functioning of the family has deteriorated and the parents have minimum control or supervision of their children. These families are described as dysfunctional with poor parenting skills, which causes a lack of communication and supervision.

Family function is poor. Therefore, they have little restraint on the children. (Police4)

I think it is because of the family […] a poor family support system. Their parents usually have no control over their children. (Worker8)

The first time he went to counselling, he did not know what to say to his father. (Health2)

The mother and son had not communicated for a long time. (Health3)

#### Passive parents

In some extreme cases, the parents have given up on their children and do not want to take part in their lives anymore. Some young people leave the reform education institution and find that they have no home to return to.

It is not uncommon that the child leaves reform school and finds that his family has disappeared. (Worker6)

Most of the parents do not even show up at school (if you call them) since they have had enough. (School2)

These dysfunctional parents will say to me “Don’t call me again,” or “Why don’t you lock him up?”, or “Let society punish him”, or “He won’t listen! I have tried everything” (School3).

A lot of parents have given up on their children. Therefore, they will say something like: “Lock him up! Beat him hard!” (Police4).

His father is used to having the court as a “stick” and when the daughter exited the facility and began to misbehave, he would say “let’s put her back in the facility” (School4).

The parents feel powerless over their own children. They will end up asking us, or the police, to step in and frighten the child, making their children behave. (Police4)

A family, in fact, becomes dysfunctional because of the social, education, and judicial departments’ deeds. (Advo2)

#### Substance using parents

The practitioners mention that adolescent clients tell them that their parents or family are the sources of their substance use habit. Some of the young people grew up witnessing their parents using illicit substances and had access to drugs from their parents and parents’ friends. In these cases, the parent might lie to the practitioners about their child’s behaviour to avoid intervention.

Her mother’s boyfriend is a drug dealer. Therefore, she got the drug from her mother’s boyfriend. (Health1)

The parents are drug dealers. Some are in prison. (Worker1)

Her father is a dealer. That is why I thought her placement in an institution is a good thing. (School4)

If their family members are using drugs, how is it possible for him not to use? (Worker6)

When we are having consultation sessions with the minors, they will tell me that their parents are using drugs. (School3)

#### Active parents

Some parents are willing to deal with their child’s drug use and actively participate in the process. If they are provided with resources, they are frequent users of the services.

### Attitudes of the practitioners

Workers’ attitudes to family work vary between focusing on adolescents (adolescent-centred) and agreeing to work with the family as important measures.

#### Adolescent-centred, not family-centred

In the current system, the support system targets young people with little attention to the parents or families. Ten of the 28 sources mentioned that their clients are young people, not their parents. Sometimes they feel a conflict of interest in working with parents. In addition, the experience of practitioners who try to contact or involve parents is usually described as exhausting, frustrating, and discouraging. All of the above might discourage the practitioners to work with families.

We actually have limited chances to work with parents. (Worker5)

If you say parents, it is probably during interviews with the young people when we meet the parents. Follow-up contact with parents is rare. (Police2)

Our focus will be talking to the students. As for the parents, they probably leave after the course. We do not have a chance to discuss the course with parents. (Probation2)

It is really difficult to try to change their parenting style and their beliefs. (Health2)

Some of the practitioners’ views is that working with the family is only effective when the family is highly functional and resourceful. The practitioners are pessimistic about the possibility of changing the behaviour of parents. Others consider that adolescents might be better off without their parents around. Still, others think that, although it is good to work with family, it is not very effective in reducing the drug use of the adolescent. One worker argued that she does not agree with family preservation, which does not work in many cases.

Many NGOs or organizations will exclude families with the excuse that the family is dysfunctional and the cause of adolescents being delinquent. (Advo2)

My opinion is that not every case needs to focus on family. Our middle home is for children who are not suitable to stay in families. (Worker6)

#### Agreeing that the parent’s role is important

One probation officer (*Probation2*) mentioned that if parents accompany their children to attend court meetings and cooperate with the court decisions, such as placement in the rehabilitation institution, treatment works better. Another worker mentioned that their experimental project assigned parents a social worker who will work closely with the adolescent’s social worker to improve family communication. This intensive contact with parents has shown some effect on changing the family atmosphere (worker9).

The power of authority is important. However, parents are still important in terms of their parenting role. Sometimes we hope that parents work with us, not ignoring or lying about their children’s misbehaviour. (Probation2)

I will personally spend more time with the family talking about their opinions of the adolescent’s behaviour and asking them what they have done about it. How do they communicate? (Worker5)

If an adolescent needs to cut off their drug-using peers, parents might need to make a lot of adjustment to their lives, including moving.

We will suggest that the parents, if it is possible, move to another county for their children’s rehabilitation services. Sometimes the parents will agree. (Health2)

### Working with families

Commonly used strategies include three tiers of work. Firstly, workers sometimes but not often will make a home visit. Secondly, they use court support, resources referrals, and substance information sharing as approaches to support the family. Finally, family intervention services are provided as parent meetings, parent counselling, and voluntary parenting courses.

#### Making contact

Making contact is the first step to work with family. Most of the practitioners use telephones. For workers in a police station, they will have the chance to accompany the parents and adolescent during police interviews. If cases are referred to NGOs for child welfare investigation, social workers will make a home visit.

The school will only call the parents if their child is in trouble. (School2)

During the home visit, we will observe the family situation. Of course, we will phone the parents first to make an appointment. Through watching their interaction, we will evaluate whether we can work with the family or not. (Worker6)

From time to time, I will make contact with the family, call them, and through conversations, make them understand how the adolescent is doing. (Health2)

#### Supporting the family

A juvenile court can refer the family to social welfare during the investigation of the case. If the adolescent is not a student, the case will be referred to the juvenile counselling committee caseworkers. During the first contact, the workers will try to listen to the parents and help them to review their past conduct and reflect on how the parents’ reactions might impact the adolescents. The aims are twofold: building a relationship with the parents and supporting the parents so that they can support their children during the process.

The child and the parent might have a lot of questions. If we can attend the hearing, it allows us to understand the whole case and procedure. If the judge says something, we will kind of translate and explain it to them. (Worker5)

In addition, supporting the family also involves finding resources, such as welfare resources, to address the family issue. Current rehabilitation clinics require guardians’ consent and accompanying their children. Therefore, many practitioners mention that they have referred medical resources for parents to take their children to rehabilitation clinics or rehabilitation facilities. Sometimes, the workers will accompany parents and adolescents to the clinics. In some cities, government funding will support rehabilitation costs (Health1).

#### Family intervention

If practitioners have successfully built a relationship with the parent, the parent might contact the worker for advice. The worker can then plan treatment or intervention. Practitioners will take any opportunity to meet parents to discuss their children’s situation, including in the court’s waiting room or during a reform education institution’s open day. In these unstructured conversations, the practitioners might take this opportunity to inquire about the child’s behaviour or the interaction between parents and child. In some cases, the worker has the chance to give feedback to the parents and provide a different perspective about the child’s misbehaviour:

If the parents want to do something but they do not know how, they will tell us a lot about what is bothering them. And we can discuss what can be done (Probation2).

Two of the workers in a non-profit organization mentioned that they recently began to provide free parent counselling for their clients’ parents. In one specific juvenile tribunal, mandatory pre-trial parent counselling was provided with private funding.

We provide free counselling for parents to talk not only about their children’s problems. We believe that parents need to work on their own problems before they can help their children. (Worker6)

Recently…we realized that a lot of our parents had tough childhoods. We did a lot to deal with their own issues because if they get better they will treat their children better. (Worker9)

Our court did not order mandatory parenting education afterward. Instead, we arranged (pretrial) psychological counselling (for parents) and we told them that the court will pay and to be sure to attend. (Probation2)

Other treatment services, such as family support groups, parents and children groups, parent services, and family meetings, were mentioned. Due to the restricted budgets and resources, statutory services are not available for every family in need.

Parents and children’s group activities are mainly designed for them to accomplish tasks or to take courses together. (Worker5)

The social workers in the juvenile police unit will contact the parents and ask them to come with their children for meetings in the police department. (School3)

These strategies are usually therapeutic and empathetic, and not about blaming the parent for their failures.

### Problems in the field

Practitioners mentioned several problems. First, they feel the mandatory sanctions for both children and parents to be harmful. The treatment might cause conflicts and tensions between the system, adolescents, and their families. The common experience of the family is to be blamed for their children’s misbehaviour again and again. Sometimes, the parents are blamed for being unqualified. This hostile atmosphere in the system usually makes working with parents more difficult. Secondly, even if they want to do more work with the family, resources are scarce.

#### Juvenile justice is considered to be a burden and sometimes harmful

It is frequently mentioned that the current juvenile treatment might hurt the parent-child relationships. The Juvenile Justice Act provides parents with the opportunity to be involved in the judicial process. It means that parents will be summoned to court or asked to accompany their children to meet the probation officers. However, in practice, this might be a burden to many families who are struggling to survive. It might worsen the relationship between parents and children and between parents and the judicial system because the process might create more conflicts rather than restoration. The judicial process increases the burden on parents who are often socioeconomically disadvantaged. One probation officer mentioned that the parents have to pay for corrective education and sometimes the parent will ask the adolescents to work to repay the money after they leave the institution.

The first reaction of the parents is to blame the children for wasting their time, making them have to go to the hearing and to take courses. (Worker1)

If the parent has encountered negative emotions in the juvenile court, they might take this out on their children. (Worker5)

Most of our treatment is asking them to come to the agency for the service, not bring the treatment to their home. (Health3)

After they finish the corrective education, our adolescents have to find a job and make a living. And maybe they have to pay for the NT$30,000 or so for their correctional education because their parents think that they owe the money to them. (Probation2)

#### Mandatory parenting education is considered ineffective

The parenting courses are supposed to “enhance their capability in performing their parenting functions” (Juvenile Justice Act Article 84). Parenting courses are designed as the last resort when protective measures are “difficult to yield desirable results” rather than as part of integrated treatment. Therefore, parents will see parenting education as punishment and not as a resource for their problems.

Parenting education, in my opinion, is punishing the parent. And 90% of the parents will complain: “I am not the one who makes mistakes. Why do I have to go to classes?” (Worker 6).

They feel: “It’s my son who uses drugs. Why do I have to take classes? Why am I the one punished?” (SW1).

Parents think that it is the children who need counselling, not themselves. (Probation1)

Since the parents do not attend courses voluntarily, it is hard to make influence on their behaviours. In addition, group courses are not designed for individual problems.

Because it is mandatory, you have to come. But in fact, it (parent education) is not effective, because they are punished by being in the class. Their mind might be absent. (Probation1)

Those with poor parenting skills basically will never turn up. (Worker6)

Some parents desperately need to reconstruct their parenting skills but they are those who will never follow court orders (Probation2).

Parents would say “I work all day, I am a labour worker. I’m exhausted after work. And I need to go to parent education at night?” (Worker5).

#### Placement or detention makes it more difficult to create attachment to family

Detention or corrective education will separate children from parents which might further endanger already distant relationships.

In corrective schools, you will find that the parents of a lot of the children from Hualien or Taitung Counties seldom visit, as visiting time is limited and they have to leave home early or spend the night in Hsinchu the day before visiting (Worker6).

If the court rules for corrective education, the juvenile will be sent to one of three facilities at Taoyuan, Changhua, or Chengzheng High School. Since Taoyuan and Chengzheng are all boy facilities, the girls will be sent to Changhua. (Worker6)

### Practitioners’ feedback

After the author presented the current practice and obstacles of family work to the focus group, the participants agreed that enhancing family functioning to be a key aim of treatment. The state should assist parents to rebuild a functional family, not replacing the family. More focus should be on the prevention of adolescent drug use and other misbehaviour by family development programmes at an early stage, by targeting vulnerable families or high-risk families. Secondly, at the municipal government level, social welfare departments and juvenile counselling committees should develop family-centred programmes by expanding more family projects and improving staff training. The juvenile justice system, including the courts and correctional education facilities, can draw lessons from the welfare system, which provides family preservation and community re-entering services for placed adolescents [61, 62]. Juvenile tribunals and courts in Hsinchu City and Kao-hsiung City have begun pretrial treatment by providing family consultation and family group courses with the support of local hospitals, and NGOs. Their experience should be researched to develop more effective family treatments.

Workers from NGOs proposed that family services should target disadvantaged families who will benefit most from the programme, regardless of whether they are involuntary clients or not. Youth social workers, including workers in juvenile consultation committees, should be trained to work with involuntary clients. It is also suggested that suitable services for the family should be based on evaluating the adolescents’ family and community environment. Therefore, practitioners should develop an outreach work model.

## Discussion

The results show that practitioners often hold negative views on the family encountered, which might discourage them from including family in work. Current contact and cooperation with the parents still fall into adolescent-centred practice. It is found that contacts and dialogue usually are random and unstructured, suggesting that only few family members might have the opportunity to speak to the practitioners. On the other hand, it is pointed out that informal contacts sometimes work better than formal meetings since the suppressive legal settings often exclude the parents. Furthermore, parents who were ordered mandatory parenting education usually felt they were being punished by the Juvenile Justice Act for their children’s failure to make progress under protective measures.

Comparing the above findings with foreign literature, I discuss three key themes which might hinder the ability of practitioners to enhance family work: the myth of parents being involuntary clients; the obstacles of developing a family project; and the argument between professional-centred and family-centred approaches.

### Are family voluntary or involuntary clients?

Many practitioners perceived the adolescent’s family as involuntary clients and need statutory family intervention to engage their involvement that legitimates the mandatory parent education. However, it has been found that family intervention can be used during the case investigation, pretrial investigation, or after the verdict when it is voluntary. It coincides with the study in New South Wales where Trotter and colleagues [56] found that among the 91 families offered a six-step family intervention when the adolescents were under community supervision or after release probation, nearly half (45 families) agreed to participate and 31 completed 6–10 weeks intervention. It suggests that when the participation of the family is voluntary, family intervention can be an option for many families.

### Obstacles to developing family projects

In terms of the practitioners’ attitudes, it is interesting to find conflicting and contradictory views. NGO workers are more willing to engage in family work and embrace the family preservation principle. On the other hand, government practitioners are more focused on the adolescent-centred work model, leaving the family work for others. Unfortunately, while adolescents are often receiving synchronous services from different agencies that disproportionally focus on post release, parochialism might cause service gaps for outreach work with families of delinquent and adjudicated youths. For example, in the action study of the parenting support outreach programme (2007 to 2014), the Ministry of Education excluded delinquent and adjudicated youths [42, 63]. Since family-included practices need a collaborative approach among different professionals, which is often lacking in adolescent substance use intervention [63], the absence of human or financial resources seems to inhibit the motivation and willingness of practitioners to develop family projects [63].

### Professional-centred? Or family-centred?

The current family work strategy stresses the provision of services to adolescents or parents separately. Most of the approaches adopt a general system theory that emphasizes changing family interactions by relationship building and resource referral [64]. The practitioners make less use of clarification, problem-solving and cognitive behavioural skills, which is similar to what Trotter and Evans found when observing youth probation interviews with adolescents [65]. It seems that practitioners usually focus too narrowly on the harm from drug use rather than the broader health needs of the adolescents and their families. Compared to the New Zealand FGC, which involves the parents and children in the decision-making process, or the New South Wales six-step family collaborative work, current family work in Taiwan fails to involve the parents in defining the problems and finding solutions. Parents are asked to support and fund the treatment suggestions proposed by practitioners. It seems that family work in current practices has not transformed from professional-centred to a family-focused or family-centred service model. Drawing lessons from the child protection practice, Huang and colleagues have found that team decision-making helps build a consensus for treatment in 59 out of 98 meetings [66]. After team decision-making, workers report that parents and families are more likely to cooperate with treatment plans. This suggests that family work can be provided as an integral part of a drug-using adolescent’s harm reduction interventions.

This qualitative study is exploratory and descriptive. The interviews were conducted in northern Taiwan and were limited to the experiences and views of 28 voluntary participants. As such, the practices in other parts of Taiwan might vary with the social resources available. In addition, their description of parents was limited to their clients in the field, excluding those families with enough resources to access private clinics or therapists. Although measures were taken to reduce social desirability bias, such as open questions and confidentially, the author is fully aware of the possibility of overestimating the extent of family programmes since those who willingly participated in this study might be those who more actively desire to impress the researcher by their good work.

## Conclusions

Consistent with previous research findings, this study confirmed that the family of drug-using adolescents are important partners to youth practitioners. Working with these families is a common practice that involves making contact, supporting the family, and in some cases providing family interventions, such as parent counselling or psychoeducation. Since most practitioners have an adolescent-centred work principle, their work with parents falls into professional-centred or family-allied work models. Unlike Family Group Conferencing (New Zealand) or collaborative family work (New South Wales), which empowers the family by involving them in the process of problem identification and prioritizing, goal setting, and collaborative decision making, our current practices stress improving the family interaction and strengthening parenting skills. Unfortunately, the judicial process consists of coercive and mandatory measures which might undermine the already intense and vulnerable relationships among the adolescents, their families, and practitioners. It is also found that the delivery of family programmes is constrained by insufficient human and other resources. Although not every family is suitable for family intervention, the process of engaging and evaluating the family has been beneficial for practitioners in the planning of an upgraded treatment strategy. It is therefore suggested that resources be reallocated to include parent counselling and intense treatment at earlier stages, instead of mandatory parental education in later stages.

## Data Availability

The datasets used and/or analysed during the current study are available from the corresponding author on reasonable request.
